# Animal Models for HIV Cure Research

**DOI:** 10.3389/fimmu.2016.00012

**Published:** 2016-01-28

**Authors:** Benjamin B. Policicchio, Ivona Pandrea, Cristian Apetrei

**Affiliations:** ^1^Center for Vaccine Research, University of Pittsburgh, Pittsburgh, PA, USA

**Keywords:** HIV, SIV, FIV, animal models, non-human primates, humanized mice, cure, viral reservoir

## Abstract

The HIV-1/AIDS pandemic continues to spread unabated worldwide, and no vaccine exists within our grasp. Effective antiretroviral therapy (ART) has been developed, but ART cannot clear the virus from the infected patient. A cure for HIV-1 is badly needed to stop both the spread of the virus in human populations and disease progression in infected individuals. A safe and effective cure strategy for human immunodeficiency virus (HIV) infection will require multiple tools, and appropriate animal models are tools that are central to cure research. An ideal animal model should recapitulate the essential aspects of HIV pathogenesis and associated immune responses, while permitting invasive studies, thus allowing a thorough evaluation of strategies aimed at reducing the size of the reservoir (functional cure) or eliminating the reservoir altogether (sterilizing cure). Since there is no perfect animal model for cure research, multiple models have been tailored and tested to address specific quintessential questions of virus persistence and eradication. The development of new non-human primate and mouse models, along with a certain interest in the feline model, has the potential to fuel cure research. In this review, we highlight the major animal models currently utilized for cure research and the contributions of each model to this goal.

## Introduction

Human immunodeficiency virus (HIV) infection in humans induces massive and continuous depletion of CD4^+^ T cells, resulting in immune suppression, which, in the absence of antiretroviral therapy (ART), culminates with the development of the acquired immunodeficiency syndrome (AIDS) and death in all HIV-infected patients. As such, the burden of the HIV epidemic, which spreads unabated such that for every HIV-infected person, two new people become infected, calls for a cure for HIV (1).

The advent of ART is one of the most prominent accomplishments of modern medicine, providing relief to HIV-infected patients through effective suppression of viremia and drastically improving their quality of life. However, life expectancy is not fully restored in HIV-infected patients on ART (2). Furthermore, ART requires life-long adherence, thus preventing effective treatment from being delivered in a sustainable way to all in need; is associated with short- and long-term toxicity; does not completely restore immune integrity; is not curative; and does not eradicate HIV-1 from the infected patients. Studies have shown that residual HIV persists virtually indefinitely, even in patients receiving ART, and that this persistent reservoir virus is replication-competent (3). The infected cells that form the reservoirs, such as resting memory CD4^+^ T lymphocytes (1, 4, 5), are refractory to ART and are invisible to HIV-specific immune responses (6–8). Moreover, the anatomic sites of the reservoirs are diffuse, with multiple tissues containing latent virus, including brain (9, 10), adipose tissue (11), spleen, lung, and other organs (12, 13). To further complicate the matter, unconventional cell types besides resting memory CD4^+^ T cells (such as T follicular helper, T memory stem cells, and T regulatory cells) have been identified as being latently infected (14–17). It has also been reported that non-T cell innate cell sets may contribute to the latent reservoir, such as monocytes/macrophages and dendritic cells (18–25), but these innate reservoirs are controversial (26). The tissue and cellular complexity of the viral reservoir results in a minimal impact of ART on the latent reservoir, even in patients receiving prolonged ART for over a decade (27–30). Removal of ART from treated patients systematically results in viral rebound, even in cases when therapy is initiated very early after infection (28, 31, 32). Rebounding virus may reseed the virus reservoirs and may carry drug resistance mutations (33, 34). Therefore, due to these potentially daunting effects of ART cessation, the current paradigm is that once initiated, ART should be maintained for life (1).

In addition to these limitations of ART, the low levels of residual viral replication observed in HIV-infected patients receiving therapy are most likely the main reason for the residual immune activation and inflammation observed during infection (35). Immune activation and inflammation are associated with comorbidities, accelerated aging, and mortality (36, 37). These issues, along with a limited availability of invasive samples from multiple potential reservoir sites necessary for a proper characterization of the reservoir, significantly impact our ability to cure HIV.

Multiple lines of documented evidence unequivocally support the fact that ART alone cannot cure HIV infection, which are as follows: (i) ART intensification does not impact the viral reservoir (27, 38, 39); (ii) the functionally cured “Mississippi baby” (40), in which a very aggressive therapy was initiated very early during infection and was maintained for 1.5 years, experienced a massive virus rebound after controlling the virus for over 2 years following cessation of ART (29); (iii) short-course ART in acutely infected subjects had a minimal impact on the reservoir (30); and (iv) cytotoxic T lymphocytes (CTLs) are necessary to eliminate the virus in patients receiving ART (41). Nevertheless, a small fraction of patients in which ART was initiated very early during infection and maintained for a long period of time (largely exceeding the half-life of the major reservoir cell populations) was reported to be able to robustly and persistently control viral replication at the cessation of ART (42, 43). Yet, since these posttreatment controllers only represent a small fraction of patients and the correlates of virus control in these patients are not completely understood, there is consensus in the field that the strategies for virus eradication should involve therapeutic approaches that go beyond ART (1).

In the aftermath of the reports of the cured HIV infection in the “Berlin patient” (44), cure research focused around developing a “sterilizing cure” (i.e., aimed at the complete elimination of the virus from the infected patient). Yet, it soon became clear that dissecting the key factor driving HIV cure in the “Berlin patient” (i.e., stem cell transplantation, radiation, immune therapy, or graft-versus-host disease) is very challenging. Furthermore, studies revolving around the cure in the Berlin patient emphasize the complexity of this process and suggest that an intricate action of multiple factors may have led to the positive outcome in this patient. This was seen in the “Boston patients,” two HIV-1 positive men that received hematopoietic stem cell transplants from donors with wild-type-CCR5^+^ cells in an attempt to provide a sterilizing cure for HIV (45). While in the initial posttransplant stages (when patients were still on ART), there was no evidence of residual infection, both men exhibited rebound of virus after ART interruption (46). This clearly suggests that a cure approach might be a difficult enterprise, and therefore, a more realistic alternative to the sterilizing cure is the development of a “functional cure,” whereby the viral reservoir is reduced enough to permit cessation of ART without risking viral rebound (40, 45).

All these various approaches toward the HIV cure cannot be directly tested in humans without major risks, making imperative for cure research the use of animal models that mimic HIV infection. In addition to providing *in vivo* systems that enable detailed studies of the multiple anatomic reservoirs through invasive sampling, the animal models of cure research also permit refining the system in order to deconvolute different components that are largely intricated in humans, which prevent our complete understanding of the correlates of viral control. Finally, animal models permit interventions aimed at depleting various arms of the immune system or specific immune cell populations that might drive formation of the reservoir or enable virus persistence. Altogether, use of the animal models appears to be mandatory for the very complex field of cure research and has the potential to significantly advance the field.

The most commonly used animal models for cure research are non-human primates (NHPs), humanized mice, and to a lesser degree, felines, each infected with their respective immunodeficiency virus. Though none of these models exactly match HIV infection in humans, each model can be tailored to address the key specific questions of cure research. This review will present the currently available animal models for cure research, along with their advantages and disadvantages (Table 1), and will focus on the contributions that each of these models have made to the cure field.

**Table 1:** **Advantages and disadvantages of the major animal models for HIV cure research**.^a^

	NHP/SIV or SHIV	Murine/HIV	Feline/FIV
Sample size	+++	+	++
Anatomy compared to humans	Similar	Different	Different
Similarity of virus to HIV	Different	Same	Different
Infection characteristics compared to human/HIV	Similar	Similar	Different
Availability for experimental infection in controlled conditions *vis-à-vis* route and dose of virus inoculation, drug regimens	Yes	Yes	Yes
Ability to deplete arms of immune system	Yes	Yes	Yes
Outbred	Yes	No	Yes
Major surgery required to generate the model	No	Yes	No
Development of graft-versus-host disease	No	Yes	No
Reservoir comparison to human/HIV	Similar	Similar	Different
Cost to maintain	+++	++	++

*^a^Fauci and Desrosiers (47), Evans and Silvestri (48), Del Prete and Lifson (49), Akkina (50), McDonnel et al. (51), and Apetrei et al. (52)*.

## NHP Models for Cure Research: Strains, Systems, and Limitations

Non-human primates are the most widely used animal models for AIDS research due to the variety of disease states that can be induced in various NHP species by a plethora of simian immunodeficiency virus (SIV) strains (Table 2). Studies in NHPs helped define key paradigms of HIV infection pathogenesis, have been used in testing various therapies, and are essential for the development of AIDS vaccines (53, 54). The NHP model of AIDS is one of the best animal models for infectious diseases, as it shares a lot of key features with HIV-1-infected humans, including anatomy, physiology, immune system, infectious agents, and susceptibility to antiretroviral treatments (55). Furthermore, due to their large size, NHPs allow for the frequent collection of relatively large volumes of samples.

**Table 2:** **Different NHP/SIV models used in cure research**.^a^

Non-human primate species	SIV strains	Acute VLs	Chronic VLs	Spontaneous elite control?	Rapid progression (frequency of RP)	Control with conventional ART	Use of NNRTI	Chronic immune activation
Indian rhesus macaque	SIVmac251/239	10^7^–10^9^	10^4^–10^7^	Yes (for specific MHC types)	30–40%	Requires complex combinations	No	Yes
	SIVsmm	10^7^–10^8^	10^2^–10^5^	Yes (for specific Trim genotypes)	No	Yes	No	Yes
	SIVagmSab	10^6^–10^9^	<3–100	100%	No	No data	No	No
	RT-SHIV	10^5^–10^8^	10^3^–10^6^	Yes (for specific MHC types)	No	Requires complex combinations	Yes	Yes
Chinese rhesus macaque	SIVmac239	10^5^–10^7^	10–10^5^	Yes (~33%)	No	Yes	No	Yes
Pigtailed macaque	SIVmac251/239	10^5^–10^9^	10^3^–10^6^	Rare	30–40%	Requires complex combinations	No	Yes
	SIVsmm	10^5^–10^9^	10^4^–10^7^	No	>75%	Requires complex combinations	No	Yes
	SIVagmSab	10^7^–10^9^	10^4^–10^6^	No	30–40%	No data	No	Yes
	RT-SHIV	10^4^–10^7^	10^3^–10^5^	Rare	>75%	Yes	Yes	Yes
Cynomolgus macaque	SIVmac251/239	10^6^–10^8^	10^2^–10^5^	Yes (~30%)	No	Yes	No	No data

*^a^Haase (56), Lackner and Veazey (57), Demberg et al. (58), Horiike et al. (59), Pandrea et al. (60), Ma et al. (61), Uberla et al. (62), North et al. (12), North et al. (63), Kauffman et al. (64), Monceaux et al. (65), Ling et al. (66), Ling et al. (67), Klatt et al. (68), Dinoso et al. (27), Canary et al. (69), Hirsch et al. (70), Mandell et al. (71), Kristoff et al. (72), Ambrose et al. (73), Shao et al. (74), Kearney et al. (75), Kline et al. (13), Benlhassan-Chahour et al. (76), Mannioui et al. (77), Sellier et al. (78), Reimann et al. (79), and Budde et al. (80)*.

Administration of SIV to multiple macaque species typically results in a persistent pathogenic infection in which progression to AIDS occurs in a similar fashion to HIV-infected patients (47). Of the wide variety of macaque species, HIV-1 research has focused on rhesus macaques (RMs) (*Macaca mulatta*), of both Indian and Chinese origin, pigtailed macaques (PTMs) (*Macaca nemestrina*), and cynomolgus macaques (CMs, the crab-eating macaque) (*Macaca fascicularis*). All these three macaque species are susceptible to infection by various SIV strains, and the disease outcome varies from elite-controlled infection, with a small rate of disease progression to AIDS-defining illnesses to a very rapid disease progression, with death occurring within a few months following infection. This variability of the disease outcomes is dependent on the viral strains used for challenge, on macaque species, and on genetic pedigree of the individual monkeys (48, 49). In addition to these different macaque species, HIV-1 research has also utilized African NHPs that are natural hosts of SIVs, such as African green monkeys (AGMs) (*Chlorocebus* genus), sooty mangabeys (*Cercocebus atys*), and mandrills (*Mandrillus sphinx*). SIV infection of these species generally does not progress to AIDS, therefore permitting comparative studies aimed at identifying the correlates of immune protection and the lack of disease progression (81–84).

Simian immunodeficiency virus infection of macaques shares the key pathogenic features with HIV infection in humans, which make macaques an ideal environment for cure research. Particularly, SIVs and HIV share the following key features of virus persistence: (i) both HIV and SIV DNA are integrated in the target cell genome (85, 86) and with a similar preference to integration site (87). (ii) Response to interferons results in transcriptional control of virus long terminal repeats (LTRs) through a bias of histone acetylation favoring HIV/SIV DNA persistence (88). (iii) Costimulatory signals can induce latent HIV/SIV without coengagement of T-cell receptors (89). (iv) Distribution of cells containing HIV and SIV DNA and RNA sequences in peripheral blood, lymph nodes (LNs), and at mucosal sites are similar in humans and macaques (77, 78, 90). (v) CTLs are ineffective in clearing infected cells long term due to resistance mutations in HIV/SIV (91, 92). These characteristics underline similar reservoir dynamics in HIV- and SIV-infected humans and macaques, respectively.

In addition to these shared features between SIV infection in macaques and HIV infection in humans, there are also several notable differences between the two models that limit the use of the macaque model and call for its improvement. Of these, the most critical for cure research are the overall higher viral loads (VLs), during both acute and chronic infections, and the natural resistance to non-nucleoside reverse transcriptase inhibitors (NNRTIs), which is a common feature of HIV-2 and SIV strains (93–95). Due to these features, SIV infection is more difficult to control with ART in macaques compared to HIV infection, requiring more potent combinations to achieve suppression (53, 96–98). This limitation can be addressed by using unadapted SIVsmm strains in macaques, which more closely reproduces the pathogenesis of HIV infection in humans and are less pathogenic than SIVmac strains (99), with the caveat that these newly developed strains are less characterized than the reference SIVmac strains.

Another notable difference between HIV and SIV infection regards their relations with the enzyme SAM domain and HD domain 1 (SAMHD1). SAMHD1 is a nuclear protein with phosphohydrolase activity that can restrict replication of lentiviruses by depleting the nucleotriphosphate (NTP) pool during reverse transcription (100). SAMHD1 is involved in HIV-1 restriction in non-dividing, resting T cells that do not support productive virus replication and is a key component of the reservoir. The viral accessory protein Vpx, which antagonizes SAMHD1 activity, has no effect on the infection of activated T cells but relieves the block to reverse transcription in resting T cells (101, 102). As such, Vpx could allow the establishment of latently infected cells, which, upon subsequent activation, would produce infectious virus that can expand the pool of infected (including latently infected) T cells. Only HIV-2 and some SIVs (notably those that are used in the macaque models) contain Vpx (103, 104). Although HIV-1 does not encode Vpx, the virus is susceptible to the infectivity enhancement provided by the SIV accessory protein (105, 106). As such, while SAMHD1 seems to play a key role in a strategy of the immune system to avoid immune cellular responses upon viral infection, it also represents a key difference between the HIV infection of human patients and the NHP animal model (107).

Rhesus macaques infected with either the reference swarm SIVmac251 or with the SIVmac251-derived infectious molecular clone SIVmac239 accurately reproduce several aspects of human HIV infections, including sustained, high VLs, immediate and progressive depletion of mucosal CD4^+^ T cells, and chronic immune activation (56, 57). As such, this model is most widely used in a wide variety of cure-based studies. SIVmac239/251-infected Chinese RMs represents another model of interest for cure research. Compared to Indian RMs, Chinese RMs have lower acute and chronic VLs, more closer to HIV-1 infected humans, and as such, SIV infection can be more readily controlled in Chinese RMs than in Indian RMs (67, 108).

Other macaque models have been also used to investigate the viral reservoirs. PTMs infected simultaneously with SIV/17E-Fr and SIV/Delta B670 represent a model of pathogenic infection, whereby the animals progress to AIDS within 3 months, with most animals developing central nervous diseases (109). This model is susceptible to tritherapy, which can suppress virus to below 50 copies/ml and was utilized to identify various anatomic sites of latently infected CD4^+^ T cells (27). It was also used to show that ART reduces both vRNA levels in cerebrospinal fluid to below the limit of detection (50 copies/ml), similar to vRNA levels in plasma, and inflammation in the central nervous system (CNS), suggesting early ART benefits levels of virus replication in the CNS and pointing to a strategy to mitigate neurological disorders that develop during chronic infection (9, 110). This model of neuro-AIDS was used for the study of the anatomic reservoir of the brain as well as the macrophage reservoir (27, 109).

Cynomolgus macaques represent another NHP model of cure research, albeit its use is more limited than that of RMs. SIVmac-infected CMs have intermediate peak VLs and low chronic VLs and, as such, are vastly easier to control with ART (76). This model is extensively used in Europe for the experiments related to cure research (11, 77, 90).

Due to genetic differences between SIVs and HIV, SIVmac strains, similar to HIV-2 strains, are not susceptible to NNRTIs (95, 98). Furthermore, SIV infection results in the selection of different epitopes compared to HIV infection. To address these limitations, SIVmac strains have been engineered to share characteristics from both types of viruses to maximize model potential. Such chimeric simian-human immunodeficiency viruses (SHIVs) were produced to include HIV-1 reverse transcriptase (RT) gene. Two RT chimeras have been produced thus far: RT-SHIVmac239 and RT-SHIVmne, SIV containing the HIV RT for the viruses SIVmac239 and SIVmne, respectively. They both overcome the NNRTI block for use in RMs and PTMs, respectively (62, 73, 111). Additionally, enhanced SHIV clones with HIV-1 Env proteins from transmitted/founder strains have been developed without passaging, thus providing a model for future studies aimed at neutralizing antibody development and testing (112).

While very useful for addressing these critical aspects of cure research, RT-SHIVs also have their limitations, including difficulty in suppressing virus replication with the same triple ART treatments used in humans (i.e., tenofovir/emtricitabine/efavirenz) (12, 73). Since most of the genome is represented by the SIVmac239 backbone, these are limitations due to the use of SIVmac.

The conventional NHP models for cure research based on the use of SIVmac recapitulate most of the characteristics of HIV-infected patients. As HIV and SIV infections are both characterized by severe immunosuppression and significant alterations of the immune responses and since studies have shown that clearance of virus reactivated from the reservoir requires functional immune responses (113) and various cure approaches requires functional CTLs to eliminate the virus reactivated from the reservoirs, we recently focused on the development of alternative models for cure research that would permit us to refine the system, in order to be able to dissect the relative impact of various interventions on the reservoir.

Thus, we developed a RM model in which functional cure of SIV infection occurs spontaneously in 100% of infected monkeys in the absence of ART (60). In this model, infection of Indian RMs with SIVsab, the virus that naturally infects AGMs in West Africa (114), results in a robust acute viral replication and a massive CD4^+^ T cell depletion. Control occurs between 2 and 3 months postinfection and is maintained indefinitely (60). Virus control is progressively consolidated, and SIVsab infection eventually becomes latent in RMs, as documented by the use of a single copy assay (61). Consequently, residual immune activation persists throughout the first year of infection before returning to preinfection levels. As a result of complete virus control, mucosal CD4^+^ T cells are slowly restored, reaching preinfection levels after 4 years postinfection (60). Furthermore, all biological and clinical markers of SIV infection (microbial translocation, immune activation, and apoptosis) are resolved and the animals serorevert by 2 years postinfection. This robust and persistent virus control can be reverted by the *in vivo* depletion of CD8^+^ cells, while restored CD8^+^ cells control the rebounding virus (60). In this model, virus control is not due to its inability to replicate, as the serial passage of the reactivated virus to naïve RMs resulted in very robust levels of viral replication, which were similar to those observed with the parental virus, clearly demonstrating that the controlled virus is replication-competent (61). This model, in which a very robust control of the virus by functional immune responses occurs without the complexity of ART, is an ideal setting for the screening of various strategies aimed at reactivating the virus from reservoirs by allowing the dissection of the effects of various reversing agents and of the corresponding immune responses without the confounding factors of ART and a weakened CTL response.

## NHP Models for Cure Research: Applications

### Use of the NHP Models to Establish Pathogenesis Paradigms

The NHP models for AIDS research decisively contributed to the establishment of key paradigms of HIV pathogenesis, and it is likely that they will play a central role in cure research. Yet, initially, the field was relatively reluctant to consider NHPs for testing therapies requiring ART coverage, because SIV and SHIV infections were relatively difficult to control with tritherapy (12, 53, 96–98), and as discussed above, residual virus replication is a major roadblock for studying virus reservoirs. However, with the renewed interest in cure research and the use of NHP models in this niche, complete suppression of VL and a noticeable effect on the viral reservoir were rapidly achieved by employing various aggressive ART regimens (9, 97, 110, 115). The original drug regimens used for the complete control of viral replication in macaques were complex and included a combination of emtricitabine (FTC), tenofovir (TDF), and raltegravir (RAL) with ritonavir-boosted darunavir and even maraviroc (97). With the advent of dolutegravir (DTG) and a coformulation of the above drugs, it was reported that control of SIVmac replication can be achieved in RMs with a tritherapy regimen (i.e., FTC + TDF + DTG) (115, 116). The ability to suppress SIVmac in RMs to the same levels of suppression seen in HIV-1-infected patients dramatically improved the prospects of cure research, allowing studies on reservoir seeding (116) or testing virus reactivation strategies (112).

One of the key obstacles for cure research is that the virus reservoir is established very early following infection. While this was postulated for quite a while based on results in vaccine studies, it was only recently directly proven (116). Thus, very rapid initiation of suppressive ART in RMs on days 3, 7, 10, and 14 after intrarectal SIVmac251 infection showed that only treatment with ART on day 3 blocked the emergence of viral RNA and proviral DNA in peripheral blood and substantially reduced levels of proviral DNA in LNs and gastrointestinal mucosa compared to treatment at later time points. Furthermore, treatment on day 3 abrogated the induction of SIV-specific humoral and cellular immune responses. Yet, when ART was interrupted after 24 weeks of fully suppressive therapy, virus rebounded in all animals, including those that were treated on day 3. However, the day 3-treated RMs had a delayed virus rebound compared to those treated on days 7, 10, and 14. The time to viral rebound correlated with total viremia during acute infection and with proviral DNA at the time of ART discontinuation. Altogether, these results demonstrated an extremely rapid seeding of the viral reservoir in RMs, during the “eclipse” phase, prior to detectable viremia, pointing to the difficulty of HIV eradication (116). Furthermore, the observed delay in virus rebound in monkeys treated very early during infection raises the question of whether or not a prolonged therapy will have a discernable impact on the size of the reservoir and on the ability to control viral rebound at cessation of ART, as reported with “Mississippi baby” (40) or the “Visconti” posttreatment controllers (43). In other studies whereby SIVmac251-infected RMs were given ART at 4 h, 7 days, or 14 days postinfection, it was shown that ART given at 4 h postinfection resulted in drastically lower virus replication and dissemination in the gut and lower plasma virus load 2 weeks following treatment compared to 7 and 14 days postinfection, further supporting a rapid establishment of the reservoir (78, 90) and calling for a very rapid therapeutic intervention in HIV-infected patients. Unfortunately, the drawback of these observations is that in the vast majority of HIV-infected patients, it is virtually impossible to initiate ART so early postinfection.

One of the very important applications of the NHP models of AIDS with major impact on cure research is the study of residual viral replication in patients on ART. Studies employing ultrasensitive viral quantitation assays reported that in treated patients, the initial rapid virus decay is followed by a very slow decay (stage III of virus decay and even stage IV decay that is likely persistent during the lifespan of the infected patient) (117, 118). A key question in the field is whether or not this detectable virus (7–10 copies/ml) is replication-competent and can contribute to reservoir reseeding. There is also a debate as to whether virus persistence seen during successful ART is the result of incomplete suppression of virus replication or residual production of virus from long-lived, chronically infected cells (27, 38, 119–121). Persistent virus replication under ART may result in the development of resistance against the drugs used to suppress the virus, requiring the addition of more toxic drugs or, in extreme cases, resulting in complete drug failure. Though these studies can be done in humans, they have major limitations as they cannot thoroughly investigate the sources of the residual virus, such investigations requiring access to multiple tissue sites. The use of the NHP model, in which we can sacrifice the animals and extensively collect multiple tissue sites and cell types, allows us to understand what the specific “sanctuary” reservoir tissues are (i.e., the locations where the virus is not suppressed), as well as the sources of virus reactivation and resistance.

Use of the RT-SHIV/NHP models represents the most efficient method to address these aspects of viral persistence under ART. Since the experimental inoculum is thoroughly characterized when infectious molecular clones are employed, experimental infections with a known inoculum followed by the administration of potent ART, frequent sampling, and extensive sequencing permit a thorough assessment of virus evolution under ART as a surrogate of virus replication. Multiple studies utilizing the PTM model infected with various RT-SHIVs have documented that viral evolution occurs under ART (75, 122, 123).

Studies in the RT-SHIV model have also shown that viral diversity does not decrease during suppressive ART and that the virus levels present prior to initiation of therapy influence the development of resistant strains (64, 75). They have also shown that upon ART reinitiation following interruption, the rate of viral decay matches the decay seen upon initial ART administration, reflecting that wild-type virus becomes integrated and emerges following ART interruption (73–75). Further studies will be needed to better characterize the fate of various viral reservoirs in patients receiving ART.

### Use of NHP Models to Test Latency Reversal Agents

The virally suppressed SIV/RM models have allowed the testing of several latency reversal agents (LRAs) to determine their ability to reactivate latent virus and assess the impact of this strategy on the size of the overall reservoir. Administration of the histone deacetylase inhibitor (HDACi) suberoylanilide hydroxamic acid (SAHA) to SIV-infected ART-suppressed RMs induced a very limited amount of virus reactivation, in spite of inducing a discernable increase in histone acetylation, indicating that repeated HDACi administrations may be necessary to see a more robust effect (67, 124). The NHP model can further be employed to test various hypotheses based on *ex vivo* experiments using HDACi. It was recently reported that administration of HDACi (particularly romidepsin, and to a lesser extent, panobinostat and SAHA) may significantly impact T-cell effector functions through either rapid suppression of cytokine production from viable T cells or through induction of selective death of activated T cells (125). As such, HDACis impaired CTL-mediated IFN-γ production, as well as the elimination of HIV-infected or peptide-pulsed target cells. It was, therefore, concluded that treatment with HDACis to mobilize the latent reservoir could have unintended negative impacts on the effector functions of CTL, which could influence the effectiveness of HDACi-based eradication strategies, by impairing elimination of infected cells (125). *In vivo* studies in NHPs are needed to confirm these *ex vivo* results.

Auranofin, a gold-based compound used to treat rheumatoid arthritis and a potential LRA, was also tested in RMs. It was reported that RM exposure to auranofin resulted in a reduction of the viral reservoir, followed by a delay in virus rebound at the cessation of ART compared to the untreated group (126). Furthermore, administration of auranofin together with the experimental chemotherapeutic agent buthionione sulfoximine (BSO) prior to ART cessation resulted in a rebound of VLs followed by control of the virus to undetectable levels and minimal immune activation for 2 years following ART cessation (127, 128).

Simian immunodeficiency virus-infected RMs were also used for testing protein kinase C (PKC) activators, such as prostratin or bryostatin. While, to date, there are no published studies reporting these results, PKC activators have been shown to have therapeutic effects at doses that are very close to toxic levels and cannot be administered for prolonged rounds of treatment without substantial toxicity or even death of the animals. Thus, PKC activators need further testing to conclude their usefulness for virus reactivation strategies.

All these experiments demonstrate that the NHP models proved their usefulness for testing virus reactivation strategies and will continue to be used for future studies. Particularly for the field of virus reactivation experiments, the use of the macaque models in which virus control occurs either spontaneously or posttreatment may be very useful for screening LRAs in an environment with functional immune responses and without the complexity of ART (52).

### Use of NHP Models to Test Transplantation Strategies

One avenue of cure research currently being explored is stem cell transplant following total body irradiation in an effort to mimic the cure seen by the Berlin patient (44). To this end, RMs were infected with SHIV and treated with ART to reduce viral replication prior to total body irradiation and engraftment of autologous hematopoietic stem cells isolated from the respective RMs prior to infection. At 40–75 days post-graft, ART was stopped. In two of three RMs, rapid viral rebound occurred, with the third RM exhibiting no plasma viral RNA 2 weeks following ART interruption (129). While the therapeutic success was limited, this study demonstrated that the replacement of the hematopoietic compartment was insufficient to eliminate host viral reservoirs. Even more important, it provided a new model for studying eradication strategies.

### Use of NHP Models to Test Strategies Aimed at Controlling Persistent Immune Activation

Chronic inflammation and immune activation were reported to be the key obstacles for cure research. Chronic immune activation persists in patients on ART (35, 130). The pool of activated cells represents one of the key sources of residual viremia (“homeostatic proliferation”) (1). Furthermore, activated CD4^+^ T cells are susceptible to infection, and increasing the pool of susceptible cells may favor reservoir reseeding, thus representing a key determinant of viral persistence (1).

The sources of chronic immune activation and inflammation in patients receiving ART are not completely known. While the virus clearly induces immune activation itself, it is also controlled with ART. Other major triggers of chronic immune activation are microbial translocation, coinfections with cytomegalovirus and other copathogens, or ART toxicity (131). Depletion of CD4^+^ T cells (through either T cell activation and apoptosis or through direct effect of the virus itself) may be responsible for inflammation (132). Thus, CD4^+^ T cell loss leads to further mucosal damage, resulting in increased microbial translocation and immune activation (133). In the pool of the CD4^+^ cells that are depleted, Th17 cells that are responsible for gut integrity and defense against microorganisms are preferentially targeted by HIV/SIV infection, resulting in the disruption of mucosal integrity and increased microbial translocation (134–136).

In addition to contributing to the virus reservoir, Tregs likewise play a role in both peripheral tolerance as well as controlling immune activation (137). Administration of Ontak, a drug that partially depletes Tregs to chronically SIV-infected AGMs, boosted both viral replication and immune activation, suggesting that Tregs may play a role in controlling the virus (138). Other studies have shown that Tregs critically contribute to the development of fibrosis and that Treg blockade with an anti-CTLA-4 antibody results in increased effector functions for CD4^+^ and CD8^+^ T cells in SIV-infected RMs (139, 140). Further studies are needed to understand the impact of Treg on the outcome of HIV/SIV infections.

Various treatments to reduce or minimize residual immune activation are being tested, and NHP models are central for such studies (141). Several studies have investigated the usefulness of interleukin (IL)-7 treatment to limit cell depletion when administered alone or with IFN-α. These studies have shown that IL-7 effectively prevented the complete depletion of CD4^+^ T cells when administered during acute infection (142). IL-7 increased proliferative capabilities and induced sustained increases of PBMC counts when administered in “clustered” doses (143); finally, IL-7 treatment prevented lymphopenia associated with IFN-α treatment while stimulating CD8^+^ CTLs against SIV (144).

Another potential therapeutic intervention is the use of IL-21 to restore/preserve T helper 17 (Th17) cells, which are responsible for mucosal integrity (135, 145). IL-21 administration to ART-suppressed RMs infected with SIVmac restored Th17 and Th22 cells, reduced immune activation in the blood and rectum, and decreased levels of CD4^+^ T cells harboring replication-competent virus (146). Also, IL-21 administration was reported to reduce the size of the viral reservoir (146). Another study showed that the administration of IL-21 combined with probiotics to ART-treated SIV-infected RMs improved recovery and maintenance of Th17 cells, contributing to reduced microbial translocation (147). Similarly, when probiotics were administered to SIV-infected PTMs on ART, levels and functionality of gut CD4^+^ T cells and APCs were increased, with a noticeable decrease in fibrosis in colonic lymphoid follicles (148).

In addition to cytokine treatment for control of immune activation, other strategies directly target microbial translocation and the potential impact on chronic immune activation and inflammation. Such strategies involved lipopolysaccharide (LPS)-sequestering in the gut with sevelamer (149). Sevelamer administration to acutely infected macaques resulted in a reduction of immune activation, inflammation, and coagulation biomarkers (72). The second strategy involves administration of intraluminal antibiotics, such as rifaximin (a semisynthetic, broad-spectrum antibiotic with poor bioavailability and is currently used to treat traveler’s diarrhea and hepatic encephalopathy) (150, 151). Rifaximin administration, in combination with the anti-inflammatory agent sulfasalazine, to acutely SIV-infected PTMs transiently and moderately improved the key parameters of SIV infection. (152). There is no information regarding the efficacy of these treatments in chronically infected RMs on ART. For these studies aimed at controlling microbial translocation-induced immune activation, PTMs are probably a better model than RMs, as gut damage is more extensive in SIV-infected PTMs. Moreover, PTMs have a higher propensity for gastrointestinal disease, even in the absence of SIV infection (153). Considering that previously published studies reported a relatively limited duration of therapeutic effect and that chronically infected patients will need to receive these therapies as life-lasting interventions, it is likely that the therapeutic applicability of these interventions will be limited. Overall, these studies point to the NHP models as critical tools for testing various treatments to reduce immune activation.

### Use of NHP Models for Vaccine Studies with Applicability to Cure Research

Development of an AIDS vaccine is considered the ultimate approach in order to control the epidemic. However, in spite of significant progress over the last decade, a vaccine against HIV is not available. In the early 2000s, it was considered that even a vaccine that does not prevent the infection but will control viral replication may be useful for controlling the epidemic (154). With the renewed interest in cure research, this concept was translated to the cure field. Vaccines that control virus replication are considered useful and will enable us to study the mechanisms of the functional cure of HIV infection. The only vaccine currently available proven to induce functional cure in RMs is based on the use of the cytomegalovirus (CMV) vectors. The concept behind the use of the CMV vectors is that infection with CMVs is benign in immunocompetent patients and results in persistent, life-long, and highly biased T cell effector memory (T_EM_) CD4^+^ and CD8^+^ T cell responses (155, 156). The replicative nature of the CMV vector and its subsequent T_EM_ stimulation, along with the fact that T_EM_ are the major T cell type found at mucosal effector sites (157), make the CMV vectors ideal candidate HIV vaccines. Indeed, rhesus CMV (RhCMV)/SIV vectors expressing SIV Gag, Rev/Nef/Tat, and Env administered to RMs prior to low-dose intrarectal infection with SIVmac239 conferred some protection to SIV challenge (156). More importantly, 50% of the RMs vaccinated with the RhCMV vectors, after experiencing an initial burst of viremia following first challenge, rapidly controlled infection through an immune-mediated partial control of SIV (158) and were functionally cured of SIV. Virus control was very robust, resulting in undetectable levels of vRNA in multiple tissues. In these RMs, CD8^+^ cell depletion did not result in a virus rebound nor did transfusion of 30 × 10^6^ cells from the cured RMs to naïve RMs result in infection. It has now been shown the RhCMV/SIV vector elicits distinct patterns of CD8^+^ T cell epitope recognition not normally seen during SIV infection (159). As such, RMs vaccinated with RhCMV-based SIV vaccines are an ideal setting for the study of the correlates of the functionally cured SIV infection.

## Use of Mice for Cure Research

Humanized mice represent an important tool for HIV cure research. Of the various types of humanized mice developed to date, the most commonly used types are severe combined immune deficiency human (SCID-hu), human hematopoietic stem cells (hu-HSC), and bone-liver-thymus (BLT) (Table 3). All three models are readily infected following HIV-1 challenge. SCID-hu mice are generated when fetal human thymus and liver fragments are implanted into mice lacking B and T cells, thus preventing rejection of the human tissue. Following implantation, the SCID-hu mouse is reconstituted with human thymocytes and naïve T cells. The drawbacks of the SCID-hu mouse model are the following: major surgery with human fetal tissue is required, the mice lack a primary immune response, reconstitution with human cells is focused around the implant, and the reconstituted cells do not efficiently spread into the periphery (50). Hu-HSC mice are sublethally irradiated non-obese diabetic (NOD) or NOD SCID gamma (NSG) that are then injected with human HSC, allowing for a more thorough reconstitution with human cells than SCID-hu mice. These mice are able to generate a near complete human immune system. The immune responses generated in this model are, however, neither robust nor HLA-restricted (50). BLT mice are a combination of SCID-hu and hu-HSC mice, whereby NOD or NSG mice are sublethally irradiated, implanted with fetal human liver and thymus fragments, and then injected with human HSC. This results in a thorough reconstitution of human HLA-restricted cells throughout the mice in most organs, with improved mucosal immunity and a more robust immune response compared to SCID-hu and hu-HSC mice (160–162). There are several limitations of the BLT mouse model, which are as follows: (i) it requires major surgery and the implantation of human fetal tissue, (ii) a long waiting period is needed until full reconstitution is observed, (iii) the development of graft-versus-host disease occurs approximately 6 months post-engraftment, and (iv) BLT mice are unable to develop high levels of hypermutated, class-switched IgG antibodies (163).

**Table 3:** **Different mouse models utilized in cure research**.^a^

	HIV/SCID-hu	HIV/hu-HSC	HIV/BLT
Methods for production	SCID mice implanted with fetal human thymus/live	NOD/NSG mice irradiated, injected with human HSCs	NOD/SCID mice irradiated, implanted with fetal human thymus/live, injected with HSCs
Timeframe needed for mouse production (prior to infection)	5–7 months from time of birth	2–3 months from time of birth	5–7 months from time of birth
Cellular composition following reconstitution	T cells	T and B cells and DCs	T and B cells, monocytes, macrophages, NK cells, DCs
Degree of colonization	Limited to thymus/live implant	Murine lymph organs and bone marrow	Murine lymph organs, rectum, vagina, gut, bone marrow
Length infection maintained	Grafts last approx. 1 year	6–7 months	>1 year
Plasma vRNA	10^4^–10^5^	Up to 10^7^	10^4^–10^5^

*^a^Traggiai et al. (164), Bonyhadi and Kaneshima (165), Brainard et al. (166), McCune et al. (167), Nischang et al. (168), Baenziger et al. (169), Berges et al. (170), and Pettoello-Mantovani et al. (171)*.

SCID-hu mice have played an important role in elucidating the characteristics of latency development during the early stages of infection. The exact mechanisms behind the development of viral latency have yet to be established, but one theory is that activated CD4^+^ T cells become infected by the virus but transition into a resting state before the host immune response or the virus itself kills the cell (172, 173). SCID-hu mice have been used to prove viral latency, which can also occur when thymic CD4^+^ CD8^+^ T cells are infected by HIV-1 and transited into CD4^+^ T cells (174). Furthermore, this model has been instrumental in the initial understanding of latently infected cells and was the vehicle utilized in the earliest latency-depleting experiments (175–178). It was shown that prostratin and IL-7, both of which are currently tested as LRAs, could activate HIV-1 from latently infected cells in the SCID-hu model (175, 176). Furthermore, it was shown that these drug-induced activated cells could be targeted for elimination with an anti-HIV envelope immunotoxin, one of the earliest “shock and kill” experiments (177).

Hu-HSC mice have significantly advanced research on understanding HIV latency. Choudhary et al. have shown that replication-competent HIV-1 persists in CD4^+^ T cells in mice receiving ART, providing an avenue for strategies targeting the viral reservoir (179). Further, the hu-HSC mouse model was utilized to test the effectiveness of a CCR5-targeting zinc-finger nuclease in controlling HIV-1 infection. Zinc-finger nuclease-expressing CD34^+^ cells were inserted into HIV-1-infected hu-HSC mice resulting in a selection of the virus for CCR5-negative cells, a reduction of HIV-1 (VLs), and a preservation of human cells (180). Hu-HSC mice were also used to test human T cell-targeted small interfering RNA (siRNA) constructs that deliver anti-CD4, -CCR5, -vif, and -tat siRNAs. It was shown that the infected hu-HSC mice which received the constructs had 30-fold lower VLs compared to the control group (181). Finally, it was shown that treatment with both anti-HIV envelope antibodies and three types of HIV latency reversing agents (vorinostat, I-BET151, and CTLA) resulted in mice controlling VLs and a decrease in the rebound frequency of the virus (182). As such, hu-HSC mice have been a useful model for identifying potential LRAs.

Unlike SCID-hu and hu-HSC mice, BLT mice are able to recapitulate a primary immune response against HIV-1 infection while remaining susceptible, thus representing the most efficient and complete mouse model for HIV studies. The BLT mouse model is readily infected by HIV-1; VLs can be readily controlled with intensive ART regimens in this model, with return of VLs upon ART cessation; and PBMCs isolated from the mice can be induced to express virus *ex vivo* (183, 184). Similar to humans and macaques, CD4^+^ T cell depletion is strain-dependent (185). BLT mice have also been used to test a CCR5-targeted RNA-interference (RNAi) treatment that provided a protective effect against HIV-1 replication (186). It was also shown that a targeted, cytotoxic anti-HIV immunotoxin therapy dramatically depleted the number of productively infected cells in various organs when combined with ART in BLT mice (187). Recently, engineered mice have been developed to contain exclusively T lymphocytes (TOM) or macrophages (MOM). As such, these models can be used to better assess the *in vivo* distribution of the reservoir (188).

Altogether, mouse models can be used to address quintessential questions of cure research, but come with a specific set of limitations, including (1) incomplete colonization of murine tissues with human cells, especially at the mucosal sites (albeit at a lesser degree in the BLT model); (2) a more limited depletion of CD4^+^ T cells in tissues (185); (3) long waiting periods for reconstitution with human cells following surgery; and (4) size limitations relative to experimental demand. This last limitation is particularly important *vis-à-vis* cure research, as the assessment of the reservoir virus and its inducibility can only be performed on large amounts of cells, normally an order of magnitude higher than what can be routinely obtained from mice. As such, the demand for more frequent and larger samples offsets the lower cost of the model by necessitating the sacrificing of a larger number of mice. Overall, the advantages of the models offset these limitations, but still necessitate the use of larger animal models for specific experiments.

## Other Models for Cure Research

Feline immunodeficiency virus (FIV) infection in domestic felines represents a large-animal model of lentiviral-induced immunodeficiency and shares similarities in pathogenesis with that of HIV-1 in humans (51, 189, 190). Furthermore, the FIV model represents the only naturally occurring model of immunodeficiency (191). Infected felines exhibit high acute viremia, depleted CD4^+^ and CD8^+^ cells, and the establishment of chronic infection, whereby the virus is controlled naturally (189). Similar to HIV-1-infected patients, CD4 counts continue to decrease until terminal illness, whereby cell-free viral RNA levels increase, immune dysfunction occurs, and opportunistic infections appear as the cat develops feline AIDS (FAIDS) (189). The FIV model had some value for the development and testing of antiretroviral drugs, particularly nucleoside reverse transcriptase inhibitors (NRTIs) (192). The FIV model can be also used to explore the potentiality of host restriction factors, as evidenced by transgenesis of the RM TRIMCyp into the cat germline resulting in feline resistance to FIV replication (193). This model has also been used to test the effectiveness of the LRA SAHA, observing increased levels of cell-associated viral RNA, one measurable point of cell-free RNA, and decreased levels of viral DNA (194).

Though progress has been made in understanding the FIV model, it has its own set of limitations, including the absence of certain accessory genes in FIV that are seen in HIV-1 (while encoding its own set of open reading frames not present in primate lentiviruses) (195); FIV utilizes CD134 instead of CD4 as the main binding receptor (196), 134, allowing FIV to infect B cells and CD8^+^ T cells in addition to CD4^+^ T cells and macrophages (197). As such, the target cells of FIV and HIV/SIV are different and with the structure of the reservoir being different, the applicability of the FIV model for cure research is likely limited.

## Conclusion

There is an acute need for an HIV cure and ART cannot provide it. Novel research with LRAs and other strategies is bringing us closer to a functional cure. Use of animal models as critical tools for cure research permits preclinical testing of the plethora of interventions that are currently contemplated for a cure strategy. The vast majority of these new strategies are tested in the various existing animal models for HIV-1. Humanized mouse models infected with HIV-1 allow for lower maintenance costs in a genetically identical model that directly mimics the human immune system but are difficult to produce, unable to be bred, and have limitations in tissue colonization and tissue demand relative to size. FIV infection of cats has only a limited applicability for cure research due to the key differences in the structure of the reservoir. SIV-infected monkeys are larger than both mouse and feline models, offering both a natural host of infection as well as a pathogenic model with a similar physiology and disease progression to HIV-infected humans but at the price of higher maintenance costs, harder acquisition of susceptible species, and the use of a different, yet genetically similar virus strains. Fine tuning of each model type, combined with novel research techniques aimed at “shocking and killing” latent virus and the development of immune-based therapies, may fuel HIV cure research.

## Author Contributions

BP, IP, and CA conceived, wrote, and edited the manuscript.

## Conflict of Interest Statement

The authors declare that the research was conducted in the absence of any commercial or financial relationships that could be construed as a potential conflict of interest.
